# Rigor in science and science reporting: updated guidelines for submissions to *Molecular Autism*

**DOI:** 10.1186/s13229-018-0249-x

**Published:** 2019-02-04

**Authors:** Joseph D. Buxbaum, Simon Baron-Cohen, Evdokia Anagnostou, Chris Ashwin, Catalina Betancur, Bhismadev Chakrabarti, Jacqueline N. Crawley, Rosa A. Hoekstra, Patrick R. Hof, Meng-Chuan Lai, Michael V. Lombardo, Cynthia M. Schumann

**Affiliations:** 1 Seaver Autism Center, Icahn School of Medicine at Mount Sinai, New York, NY 10029 USA; 2 Department of Psychiatry, Icahn School of Medicine at Mount Sinai, New York, NY 10029 USA; 3 Department of Genetics and Genomic Sciences, Icahn School of Medicine at Mount Sinai, New York, NY 10029 USA; 4 Fishberg Department of Neuroscience, Icahn School of Medicine at Mount Sinai, New York, NY 10029 USA; 5 Friedman Brain Institute, Icahn School of Medicine at Mount Sinai, New York, NY 10029 USA; 6 Mindich Child Health and Development Institute, Icahn School of Medicine at Mount Sinai, New York, NY 10029 USA; 7 Autism Research Centre, Department of Psychiatry, Cambridge University, Cambridge, CB2 8AH UK; 8 Bloorview Research Institute, Holland Bloorview Kids Rehabilitation Hospital, Toronto, ON M4G 1R8 Canada; 9 Department of Pediatrics, University of Toronto, Toronto, ON M5S 3H7 Canada; 10 Centre for Applied Autism Research, Department of Psychology, University of Bath, Bath, BA2 7AY UK; 11 Sorbonne Université, INSERM, CNRS, Neuroscience Paris Seine, Institut de Biologie Paris Seine, 75005 Paris, France; 12 Centre for Autism, School of Psychology & Clinical Language Sciences, University of Reading, Reading, RG6 6AL UK; 13 Department of Psychiatry and Behavioral Sciences, MIND Institute, University of California Davis School of Medicine, Sacramento, CA 95817 USA; 14 Department of Psychology, Institute of Psychiatry, Psychology and Neuroscience, King’s College London, London, SE1 1UL UK; 15 Centre for Addiction and Mental Health and The Hospital for Sick Children, Department of Psychiatry, University of Toronto, Toronto, ON M5T 1R8 Canada; 16 Department of Psychiatry, National Taiwan University Hospital and College of Medicine, Taipei, 10048 Taiwan; 17 Department of Psychology, University of Cyprus, 1678 Nicosia, Cyprus

There was some controversial press reporting of an article published in *Molecular Autism* by Anwar et al. [1]. In addition, at least two organizations dedicated to scientific accuracy in reporting have commented on this article, including www.sciencemediacentre.org and www.healthnewsreview.org. While the authors expressed some of the caveats of the study in their article, the subsequent reporting in the press and non-specialist community omitted critical caveats and we feel compelled to alter our guidelines to better address such issues.

A particular concern is claims about a biological test being diagnostic. Countless publications have made the point that what is observed in a selected sample of cases and controls is very far from being a diagnostic test. Drs. Paul Meehl and Albert Rosen summarized many of the relevant issues as early as 1955 [2], in response to a 1954 white paper by the American Psychological Association [3]. In summary, findings of even *very significant differences* in a given measure between cases versus controls are not *by themselves* sufficient to support the use of such a measure for diagnosis. This is especially true for rare events because for “any attempt at prediction of infrequent behavior, a large number of false positives are obtained” [4]. Even with an incidence of around 1%, autism would be considered such a rare event.

Researchers focusing on diagnostics are generally aware of these issues and reviewing such issues is (or should be) part of any good training program for clinicians and for anyone involved in translational research. While there have been many papers over the ensuing 60 years that made these points, we will review them once more in the current context.

The important terms to remember are sensitivity and specificity. *Sensitivity*, in the context of autism diagnoses, is the proportion of individuals with autism that are correctly identified as having autism. *Specificity*, in contrast, is the proportion of typical individuals who are correctly identified as typical. The best classifier identified by Anwar et al. had a sensitivity of 0.92 and a specificity of 0.84.

While these numbers are quite intriguing, the results in Anwar et al. are derived from a sample that is maximally 31 typical individuals and 39 individuals with autism. In other words, the sample size was exploratory and, most importantly, 55% of the individuals in the study have an autism diagnosis, compared to approximately 1% in the general population. Here, we consider how the same sensitivity and specificity plays out in a population sample (i.e., assuming 99% of the sample population is typically developing).

**Table:** 

Anwar et al. 2018				Percent with autism
		Actual diagnosis		
		Typical	Autism	
Predicted diagnosis	Typical	26	3	
	Autism	5	35	
	Total	31	38	55%
Simulated population sample				Percent with autism
		Actual diagnosis		
		Typical	Autism	
Predicted diagnosis	Typical	3160	3	
	Autism	602	35	
	Total	3762	38	1%

As can be seen from the Table, in a more representative sample, using the same sensitivity and specificity measures but with a base rate of 1%, *fully 95%* (602 out of 637) of individuals in the population receiving an autism label based on the screen would not have autism. In addition, under both scenarios, 8% of individuals with autism would be misclassified.

There are some scenarios where such a high false-positive rate might be acceptable, for example, where the burden is small, the outcome dire, and immediate treatment with an effective intervention is critical to changing the outcome (e.g., neonatal screening for treatable metabolic disorders). However, it would be hard to argue for such a test in autism where we have reasonable tools for diagnosis and the effective interventions are both costly and can be administered over a larger temporal window.

Moreover, this remains a most optimistic scenario. For example, we have no idea how other conditions may test using this screen and the authors appropriately call for an “assessment of the specificity of the algorithms for autism versus other psychiatric conditions.” And note that for some measures in the Anwar et al. study [1], the numbers of typical and autism subjects were lower (e.g., 21 vs 27 for the metabolite that was associated with the best classifier).

In addition, the sensitivity and specificity were chosen as the best from multiple different algorithms and from multiple different tests, without formal correction for experiment-wide error, and replication of such findings is a sine qua non for advancing any potential screen or even any scientific findings. This is especially true for a condition that is as etiologically and clinically heterogeneous as autism. Again, the authors make this clear in their Conclusions, where they note: “For future studies, we suggest firstly validation of the current findings in an independent clinical study group.” With validation in an independent sample and prospective studies of, for example, high-risk individuals, together with studies in other neurodevelopmental conditions, we may begin to have another clue into the biology of autism. But likely not a diagnostic test.

This nuance and cautious reporting by the authors was not always well reflected in reports in the press. Here are two out of several examples: “Blood test which is 90% accurate could help to diagnose autism in children by detecting early warning signs” (*Daily Mail*); and, “The most reliable of the tests is better than any other diagnostic method available” (*Newsweek*).

Many of the relevant news reports do not include any feedback from experts. When they do, they are much more balanced. *CNN*, for example, noted that “experts caution that the tests are far from becoming available clinically and that more research needs to be done.” And they include opinions from individuals not involved in the study. Dr. James Cusack is quoted as saying that, “This (study) is weakened by a small sample size, possible overfitting of data and a lack of comparison groups,” and that, “This study does not tell us how effectively this measure can differentiate between autism and other neurodevelopmental or mental health conditions such as ADHD and anxiety.”

And *CNN* goes on to quote Dr. Max Davie, a spokesman for the Royal College of Paediatrics and Child Health: Dr. Davie notes that “This is a promising area; however, this is a very long way indeed from a test for autism,” and that, “It is important that it is not adopted with too much enthusiasm. If applied to a large population, it will produce large number of false positives, causing huge worry and potential harm to children and families.”

As scientists who have worked in the field of autism research for decades and have learned the importance of always putting families first, we need to protect against the risk of scientific inaccuracy by emphasizing limitations and caution, to maximize the accuracy of public summaries of any study. *Molecular Autism* prides itself on being open access and we hope that our papers are accessible to families and to non-experts. The upshot of this must be that the Editors-in-Chief, Associate Editors, reviewers, and BMC staff do everything we can to give an honest appraisal of impact in every paper, no matter how redundant the cautions may be.

As Editors, in relation to the Anwar et al. study [1], we should have insisted that any mention of diagnostics should have been minimized in the publication and the limitations be explicitly addressed. Although the authors and the expert reviewers were clearly aware of the limitations, we should have emphasized and re-emphasized them in the publication.

As a second link in the chain we note that, in many examples of press surrounding the study by Anwar et al. [1], the text in print and online news was taken from a university press release. There is evidence that a source of exaggeration of research findings in the news can result from exuberant press releases from both universities and journals [5, 6]. Importantly, these same studies show that neither exaggerated claims nor caveats included in press releases overly influence the likelihood of news coverage, so being more cautious does not come at any real cost to the authors, their universities, or to the journal. It is the responsibility of authors and editors to work with institutional and journal press offices and ensure that caveats are clear and exaggerated claims are minimized.

The third link in the chain is that of the news organizations themselves. Ideally, these organizations should carry out critical reporting on news stories, which includes getting feedback from experts not involved in the work. In some cases, this can be done with little effort. For example, the quotes from Drs. Cusack and Davie come directly from the Science Media Centre, a group that solicits expert comments in order to help journalists understand and cover stories accurately (http://www.sciencemediacentre.org/expert-reaction-to-blood-and-urine-biomarkers-for-autism/). The Science Media Centre sends these quotes directly to the press before the embargo is lifted and journalists often use these quotes in their reports.

Additionally, the American Association for the Advancement of Science has developed SciLine (https://www.sciline.org). SciLine connects scientists and journalists to help ensure that scientific principles and evidence are conveyed accurately and in proper perspective (the name SciLine blends “science” and “deadline,” to emphasize the need for rapid, accurate responses from scientist volunteers).

HealthNewsReview.Org dissects some health claims in the popular press and commented on a report in the *Guardian* press around Anwar et al. as well. They note that “the story lifts quotes and almost entire sentences from a news release with no apparent original reporting.” In addition they remark that: “What would have helped this story considerably would be interviewing sources not affiliated with the study to clarify the limitations of these biomarkers, explain how autism is currently diagnosed, and provide some much-needed context about the novelty, availability, and clinical relevance of the biomarkers studied” (https://www.healthnewsreview.org/review/guardian-trumpets-test-diagnose-autism-wheres-evidence/).

To their credit, in response to this criticism, the *Guardian* posted a few days later a note with a statement from the UK autism research charity, *Autistica*: “This is a small, early-stage study which may explain one biological difference in autism. At this stage, the results presented are not strong enough to suggest that this method could be used for the diagnosis of autism. For example, we don’t know whether this technique can distinguish autism from ADHD, anxiety or other similar conditions. There have been many previous attempts to develop a biological test for autism. Still, the best way to diagnose autism is through clinical interview and observation, which takes into account the many features of autism.”

We are therefore instituting some additional steps in our publication process to help ensure that we minimize the gap between what is presented in our journal and what is reported in the larger community.

First, we require a description of limitations. In addition, we feel that the expectations that are universal for basic science must also be applied to participant based research. In an Editorial on genetics we noted that (1) suitable (i.e., large) sample sizes, (2) small *P* values including correction for multiple testing, (3) confirmatory replication studies, and (4) estimates of effect size are required for any such submission to *Molecular Autism* [7]; these guidelines will now be applied more broadly as detailed below. We also ask that attention be paid to press around articles. These changes are enumerated here.

1. **Limitations**
  Every article must now include an explicit Limitations section. Articles without this will not be reviewed and the handling editors and reviewers will be asked to review and evaluate this section carefully.
2. **Sample size**
  While, in the past, sample sizes of thousands seemed unattainable for genetics and then for imaging, in both cases, these have been achieved in autism. For blood and urine tests, we would expect that, in any submission to *Molecular Autism*, the discovery set have over 200 subjects and that the replication sample be of a similar size. For other studies, optimal sample sizes are not always attainable or even defined. For example, there are only modest numbers of postmortem brains for autism. Similarly, a study of a rare genetic disorder may include only a modest number of participants. We would still welcome such studies, recognizing, for example with postmortem studies, that the results with larger samples may overturn prior results as recently happened in schizophrenia [8]. Such issues would now be presented in the Limitations section.
3. ***P*** **values**
  There is a significant body of empirical and theoretical data that raises questions about the meaning of a nominal *P* < 0.05. A very large number of papers have recently addressed possible solutions to the issues of *P* values ([9], together with over 20 co-publications in the same issue; [10, 11]). Benjamin et al. [10] (which included over 70 experts) suggested lowering *P* value thresholds to 0.005. While we will continue to consider manuscripts with more modest *P* values, a frank discussion of the careful interpretation of such values should appear in Limitations. Of course, some important, negative studies will not have significant *P* values, and such studies are welcome.
4. **Replication samples**
  In biochemical, molecular genetic, cell and animal studies, there is now a universal expectation of both suitably powered studies and replication. Why should participant-based research, with the burden it necessarily places on families, and with the extreme heterogeneity of autism, be held to a lower standard? We expect to see a replication of any significant, primary finding in all manuscripts.
5. **Effect size**
  In genetics (and in meaningful clinical trials and meta-analyses), the effect sizes of the major findings are reported. We requested this in our prior editorial directed at genetic studies [7] and we now require it for all studies. Note that as data sets get larger, it is easier and easier to have lower *P* values. Whether these low *P* values are meaningful either scientifically or clinically can only be assessed by the inclusion of effect sizes for primary results. Genetic or other -omics studies with very low *P* values may have effect sizes that are simply too small to be useful. For these studies and for smaller studies, including effect size, with confidence intervals, will aid in interpretation. Another important approach is sensitivity power analyses. Borrowing from the new requirements for the *Journal of Experimental Social Psychology* (see https://www.journals.elsevier.com/journal-of-experimental-social-psychology/news/announcement-of-new-policies-for-2018-at-jesp), we ask that: “Each original empirical study with existing data should report, for its key hypothesis tests, a sensitivity power analysis” (available in the free software program GPower http://www.gpower.hhu.de/en.html), reviewers and editors may then, “ask authors to justify why an experiment only powerful enough to detect a conventionally “large” or greater effect size was run.” This is a useful and helpful approach and aids in interpretation.
6. **Press release**
  We would ask that authors carefully consider the language in any press release and consider coordinating press releases with the journal. Coordinating press releases with the journal can minimize discrepancies in message. This would also avoid the awkward situation where the journal or its Editors feel the need to respond to claims in the press.

With these steps (i.e., a section on Limitations, appropriate sample sizes, appropriate *P* value interpretation, effect size and sensitivity power analysis, replication and validation in an independent cohort, and attention to coincident reporting by author institutions), applied to all studies in *Molecular Autism*, we will ensure that we remain at the vanguard of important and impactful research in autism, while ensuring that reported findings and their potential impact are understandable to the broadest audience.
